# Bacteriospermia-Related Male Infertility: A Case Report on Diagnostic and Therapeutic Approaches

**DOI:** 10.7759/cureus.62973

**Published:** 2024-06-23

**Authors:** Gunjan Lakhe, Nancy Nair, Charu Pareek, Sarita Ugemuge

**Affiliations:** 1 Clinical Embryology, Datta Meghe Institute of Higher Education and Research (DU), Nagpur, IND; 2 Microbiology, Datta Meghe Medical College, Datta Meghe Institute of Higher Education and Research (DU), Nagpur, IND

**Keywords:** antioxidants, vitamins, antibiotics, semen culture, dna fragmentation, bacteria

## Abstract

Male infertility is significantly affected by bacteriospermia, defined by the presence of bacteria in semen. This case report aims to address the effects of bacteriospermia and its correlation with semen characteristics, sperm DNA fragmentation (SDF), and reproductive outcomes. The 33-year-old male was diagnosed with bacteriospermia caused by two gram-negative bacteria: *Escherichia coli *and *Klebsiella pneumoniae*. It was found that sperm parameters such as mobility, number, and morphology were compromised in sperm, indicating poor fertility. In addition, SDF analysis has revealed a high DNA fragmentation index (DFI), emphasizing the detrimental effects of bacteriospermia on the integrity of sperm. Antibiotic therapy and antioxidant supplements have been used as therapeutic measures to reduce the impact of bacterial infections and DNA damage caused by oxidative stress (OS). Follow-up assessments showed significant improvements in the integrity of the sperm DNA and the resolution of microbial colonization, which ultimately led to successful *in vitro* fertilization (IVF) and embryo transfer (ET), leading to a positive pregnancy outcome.

## Introduction

Urogenital tract infections (UTIs) and inflammation have been shown to affect the quality of semen and are important emotional factors in male infertility. Inflammation or infection affects male infertility in between 4 and 64% of cases. Reduced activity of sperm cells and possibly the entire spermatogenesis can be caused by acute and chronic infections and continuous inflammation [1].

Bacteriospermia is a condition characterized by the presence of bacteria in the semen, which has a significant influence on male infertility [2]. In recent years, medical and scientific communities have begun to pay more attention to the colonization of bacterial cells in male reproductive organs, cells, and fluids and the effects of bacterial morphology, activity, and fertilization [3]. There are possibilities that microorganisms in semen may have a significant negative impact on subsequent male fertility. However, it remains to understand the complex and multifaceted molecular foundations that underpin the quality of semen associated with bacteriospermia [4]. Bacteriospermia is associated with increased DNA level fragmentation of sperm and negative effects on sperm characteristics such as motility and morphology [5]. Although the precise mechanisms by which bacterial species cause these harmful effects are unknown, it is assumed that they can cause oxidative stress (OS), inflammation, and other pathogenic processes in the male reproductive tract [6].

Sperm DNA fragmentation (SDF) is an important factor in assessing male infertility because it can be induced by endogenous factors such as defective maturation or OS, or by exogenous sources such as disease conditions, lifestyle risk factors, and environmental exposures. The 20% SDF threshold is considered accurate. Clinical scenarios where SDF testing is most beneficial include unexplained infertility, recurrent pregnancy loss (RPL), varicocele, assisted reproductive technology (ART) patients, and patients with lifestyle and environmental risk factors. Treatment methods include recurrent ejaculation, antioxidant therapy, and lifestyle modification [7].

Semen culture is regarded as a crucial diagnostic tool in evaluating a genitourinary tract infection because inflammations and infections of the genitourinary tract can be causes of infertility in men. The secretions from the accessory sex organs, such as seminal vesicles, prostate gland, and bulbourethral glands, along with spermatozoa, constitute semen [8]. Normal urine flow preserves the sterility of the internal urethra. The distal urethra is not regarded as a sterile region. As a result, growing organisms from semen samples usually result from culture, many of which are considered normal genitourinary tract flora [9].

The antioxidant supplement is used for treating male infertility. These include coenzyme Q10 (CoQ10) or ubidecarenone, lycopene (carotenoids), omega-3 fatty acids, carnitine, vitamins E and C, selenium, glutathione, N-acetyl cysteine, zinc, and vitamin B12. It takes part in aerobic cellular respiration as a part of the electron transport chain and produces energy in the form of adenosine triphosphate (ATP). It is used in the electron transport chain in its oxidized state. CoQ10 is mostly found in the mitochondrial mid-piece of sperm cells, playing a role in energy synthesis. It acts as an antioxidant to stop sperm membrane lipid peroxidation. CoQ10 significantly increased sperm motility, count, and fertilization rate, according to a randomized controlled trial [10]. Lycopene is a potent antioxidant and a vivid red carotenoid pigment. It is the most effective way to neutralize free radicals and oxygen [11].

This study addresses the impact of bacteriospermia on male infertility, drawing attention to the importance of timely diagnosis, particularly in cases involving gram-negative rods (GNRs) such as *Escherichia coli *and *Klebsiella pneumoniae*. The administration of antioxidants to the patient showed positive outcomes, making OS an important factor in treating bacteriospermia. These findings contribute to the existing literature, highlighting the necessity of incorporating OS management in therapeutic strategies for bacteriospermia-related male infertility.

## Case presentation

Patient information

A 33-year-old male and a 27-year-old female visited the test tube baby centre. For five years, the couple has been trying to conceive. Their primary concern was the inability to conceive. The patient reported no significant past medical conditions or surgical procedures. No inflammation of the urinary tract has ever been diagnosed. There was no family history of infertility or reproductive issues. The patient denied any significant medical condition, urological disorders, or hormonal imbalances. He had not received any prior fertility evaluations or treatments. When taking the patient's occupational history, he had been working in an office environment, which means there was no previous exposure to any occupational hazards or toxins such as endocrine-disrupting chemicals (EDCs). Due to infertility over the past 5 years, the patient reports psychological issues by expressing his feelings of frustration and anxiety. He also noted a supportive and understanding relationship with his partner throughout the process and agreed to ART treatment.

The male patient was suggested for semen analysis, and the female partner for a complete hormonal profile. The hormonal profile of the female partner was normal in the biological range along with a mid-range Anti-Mullerian hormone (AMH) value of 2.2 ng/mL indicating that her ovarian reserve was within normal limits.

The clinical findings observed during the assessment of a male patient included: the volume of the semen sample measured at 2.5 mL, exhibiting an opaque grey colour and liquefied viscosity. Liquefaction occurred within 15 minutes. The total sperm count was determined to be 13 million per milliliter (mil/mL), with 45% showing motility. Among the motile sperm, none were classified as grade IV, 5% were grade III, 15% were grade II, and 25% were grade I. The remaining 55% were non-motile. The microscopic evaluation also showed sperm agglutination. Pus cells were present at a concentration of 2 mil/mL, while red blood cells (RBCs) and epithelial cells were absent as mentioned in Table 1.

**Table 1:** Semen parameters of male patient mil: Million; mL: Millilitre; RBCs: Red blood cells

Semen Parameters	Findings
Liquefaction time	15 minutes
Total sperm count	13 mil/mL
Motility	45%
Grade IV	00%
Grade III	05%
Grade II	15%
Grade I	25%
Non-motile	55%
Pus cells	2 mil/mL
RBCs	Absent
Epithelial cells	Absent

**Figure 1:**
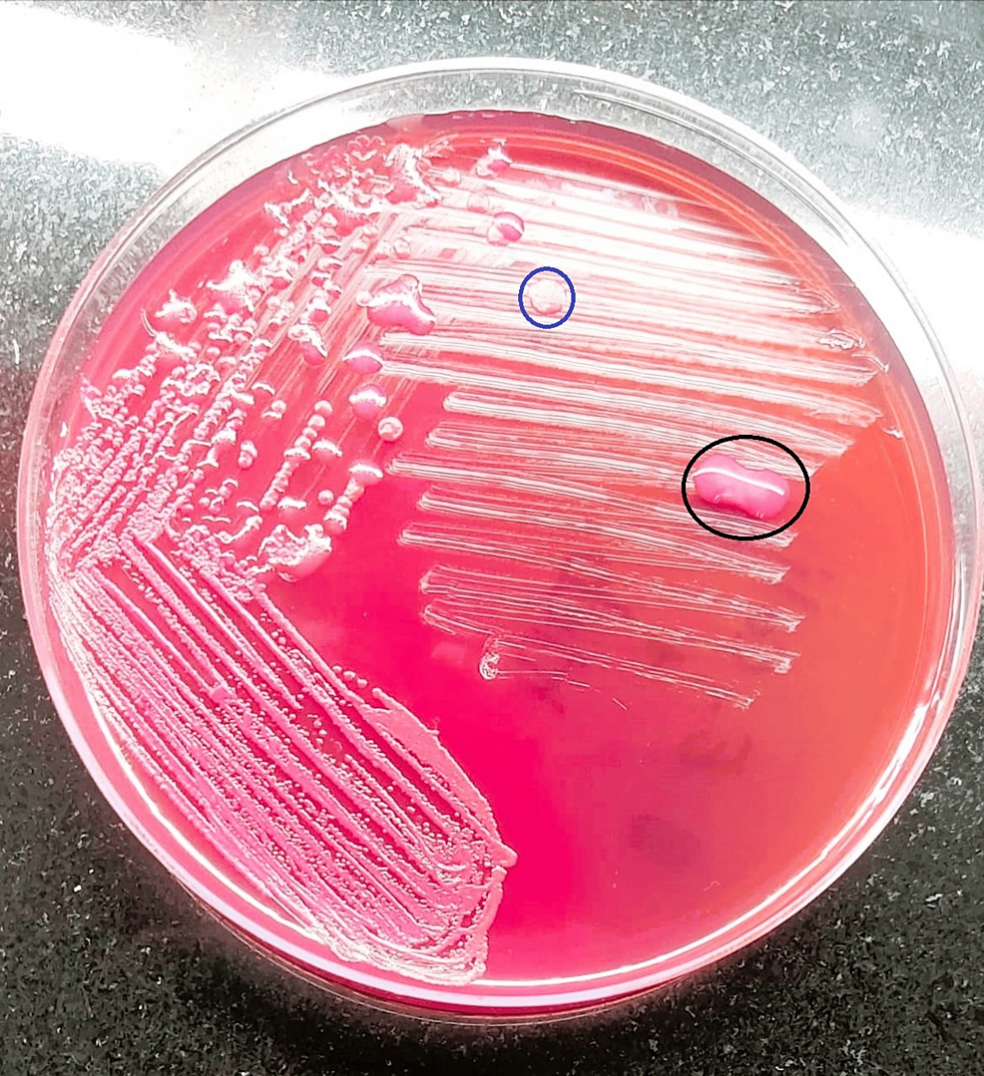
Mixed microbial growth in the semen culture The blue circle shows the isolated colony of *E. coli.* The black circle shows the isolated colony of *K. pneumoniae.*

Given the low sperm count and motility, the patient was advised to undergo further testing. Other unfavourable factors included a high number of leucocytes. Consequently, an SDF test and a semen microbiological investigation were recommended. The SDF test used the chromatin dispersion technique. The results revealed a high DNA fragmentation index (DFI) of 40%, and the microbial results indicated that the semen sample showed a positive culture of *E. coli* and *K. pneumoniae *mix bacteria grown on MacConkey agar. These were later isolated and further confirmed by the Vitek-2, an automated system for identifying bacteria and testing their susceptibility, utilizing fluorescence-based technology with the antibiotic susceptibility test (AST). These diagnostic results indicate the possibility of bacteriospermia caused by *E. coli* and *K. pneumoniae*, together with affected sperm parameters, may contribute to the patient's infertility. Figure 1 shows mixed microbial growth on the MacConkey agar plate.

**Figure 2:**
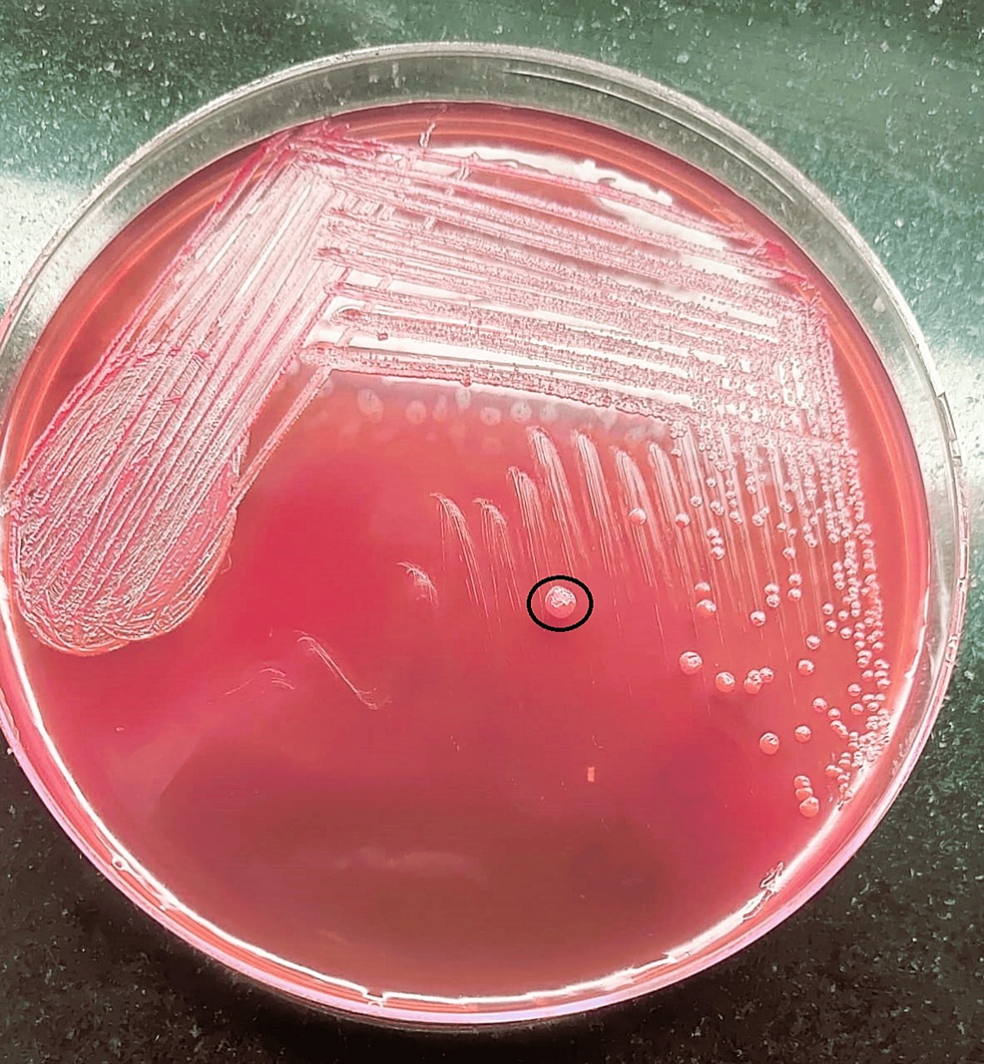
Isolated growth of E. coli The black circle shows the isolated colony of *E coli.*

Figure 2 shows the isolated growth of *E. coli *on the MacConkey agar plate.

**Figure 3:**
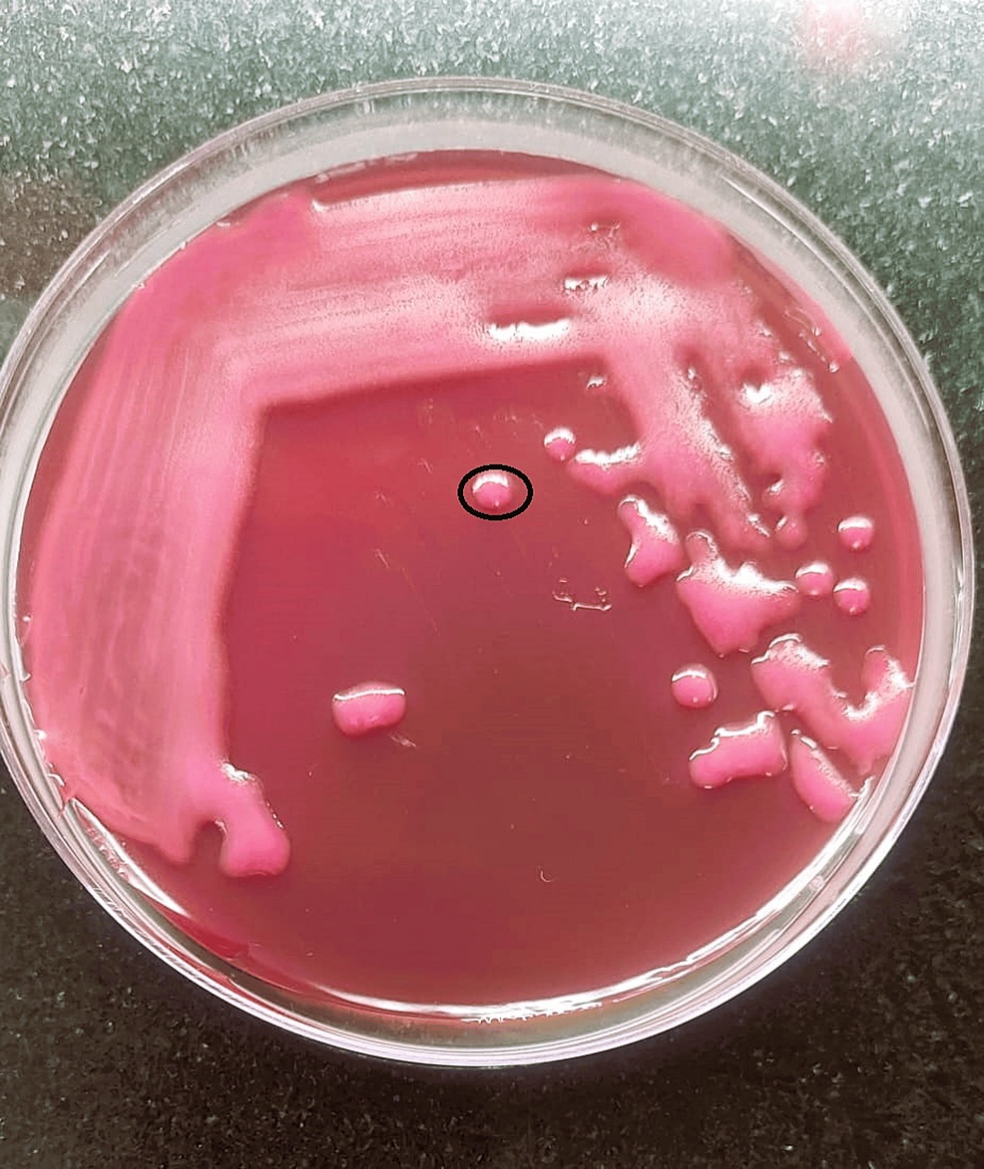
Isolated growth of K. pneumoniae The black circle shows the isolated colony of *K. pneumoniae.*

Figure 3 shows the isolated growth of *K. pneumoniae* on the MacConkey agar plate. 

Following the observation of abnormal semen analysis findings, including a high DFI and a positive microbiological culture, the patient was prescribed a regimen of doxycycline at a dosage of 100 mg twice daily for 7 days. Additionally, the patient was advised to supplement their treatment with antioxidant compounds such as CoQ10 and vitamins C and E for 3 months before a follow-up assessment. Dietary recommendations supporting reproductive health, such as a balanced diet rich in antioxidants and omega-3 fatty acids, were made. Lifestyle modifications, including regular yoga practice, meditation sessions, and early morning walks, were also recommended to enhance overall well-being.

Upon completing the prescribed regimen and lifestyle adjustments, the patient underwent repeat testing, including semen analysis, assessment of SDF, and semen microbial culture. The results indicated a normalization of parameters, with a negative finding for semen culture.

Afterward, the couple was provided with complete information about various ART, including *in vitro* fertilization (IVF) with embryo transfer (ET), with detailed explanations of potential benefits and risks associated with each option. Both individuals provided informed consent before proceeding with any further interventions.

After receiving a favourable report, the couple proceeded with another IVF-ET round with complete information regarding various reproductive treatment options, including procedures, potential benefits, and risks. Both participants provided informed consent before any further intervention was undertaken.

**Figure 4:**
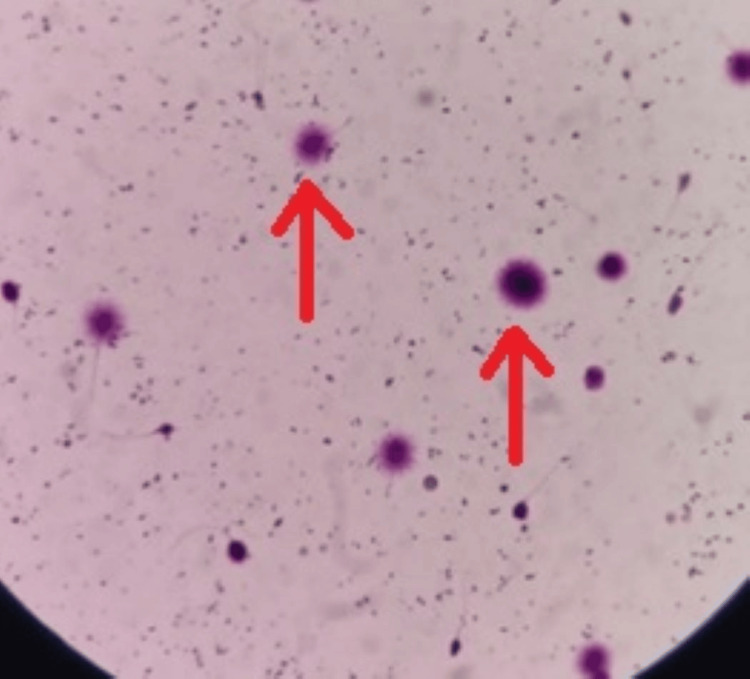
Sperm DNA fragmentation of male patient post-treatment The red arrows point to the big halos suggesting less DNA fragmentation.

Follow-up testing revealed better sperm DNA integrity as shown in Figure 4.

**Figure 5:**
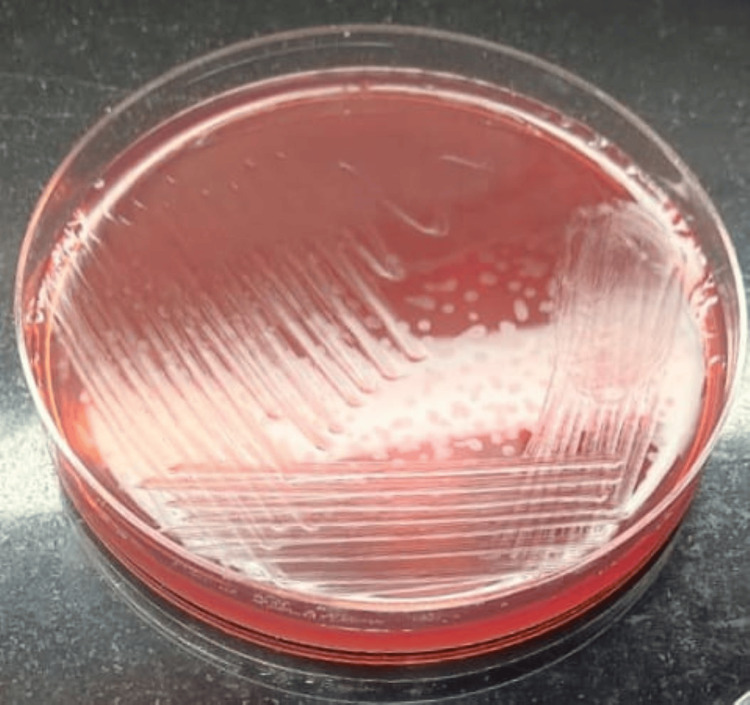
No growth in semen culture

The patient was also advised for a follow-up semen culture showing negative growth, confirming an effective treatment outcome shown in Figure 5.

The female patient's oocyte pickup (OPU) preparation commenced with a brief antagonist treatment. Before administering the gonadotropin-releasing hormone (GnRH) antagonist, follicle-stimulating hormone (FSH)/human menopausal gonadotropin (hMG) was used to stimulate the leading follicle to mature to a diameter of 14-16 mm. Human chorionic gonadotropin (hCG), along with 2.5 or 5 mg/d hMG, was administered as part of a short antagonist program to enhance ovarian function. A dose of 5,000 IU of hCG was given 36.5 hours before ovum pick-up, as the GnRH antagonist facilitates oocyte maturation.

The OPU (ovum pick-up) retrieved around 15 oocytes, 8 of which were MII, 5 MI, and 2 GV. All of these 15 oocytes were injected with spermatozoa obtained from a fresh sample of the male, and 11 of them showed fertilization with two pro-nuclei. The embryos were further cultured in media. The results are shown in Table 2. 

**Table 2:** Day-wise embryo status

Day 0	Day 1	Day 2	Day 3	Day 4	Day 5
15 oocytes	11X2 pronuclei	7X4 cell	7X8 cell	3X compaction	2X early blastocyst

Five embryos were formed and preserved for the next planned ET. Two embryos, grade 3AB and grade 3BB, were transferred in the second planned cycle followed by a positive B-hCG test of 200 mIU/mL. A constant follow-up was scheduled every two weeks to check the fetal growth rate.

Follow-up and outcome

After ET, the female partner was prescribed vitamins, CoQ10, iron, and calcium supplements. On day 14, a follow-up appointment was scheduled, and she was suggested for a urine pregnancy test (UPT). UPT test was positive, and positive pregnancy was confirmed by a 300 mIU/mL serum β-hCG examination. Two weeks later, the patient was scheduled for an ultrasound confirming the presence of a single healthy fetus growing at a normal rate. Eventually, a healthy 2.8 kg baby girl was delivered by cesarean section at 36 weeks of gestation.

## Discussion

Bacteriospermia, or the presence of bacteria in semen, can have a substantial impact on male fertility. This case underscores the significance of conducting a full diagnostic assessment, which includes semen analysis, SDF testing, and microbiological culture, to discover underlying causes of infertility. In a study, Hannachi et al. concluded that bacteriospermia can affect sperm parameters. Treatment for this ailment appears to boost sperm fertilizing potential. The given case study shows the intricate connection between bacteriospermia and SDF. The thorough diagnostic examination and diverse therapy approach shed light on the problems and potential of dealing with such situations. This study expands on the findings, discussing the consequences and the treatment's usefulness [12].

In a study, Periasamy et al. discussed the impact of different bacteria on seminal parameters, which resulted in male infertility. It is now necessary to include bacteriospermia as a factor influencing male fertility, and hence, screening for bacterial infection should be performed before treatment to overcome infertility. This verifies the research strategy on bacteriospermia and its prevalence in male infertility, as well as the usefulness of semen culture in ruling out bacteriospermia as a cause of infertility. SDF, a new indicator of sperm quality, has garnered attention due to its influence on reproductive outcomes. The case study found a link between bacteriospermia and increased DNA fragmentation, most likely caused by the bacterial infection's OS. The treatment approaches, which included antioxidant supplements and lifestyle changes, resulted in a significant increase in the integrity of sperm DNA [13].

The research conducted by Pagliuca et al. strongly supports the statement linking infection status with sperm volume, concentration, and motility. Their findings also indicate a significant likelihood of fragmented sperm DNA in affected samples. Given that urogenital infections often lack noticeable symptoms, a thorough examination of seminal samples can aid in both diagnosis and treatment, providing valuable insights for managing male infertility. However, like any case study, certain limitations should be acknowledged. Individual responses to treatments may vary, and the findings may not universally apply to all cases of bacteriospermia and DNA fragmentation. Additionally, the relatively short duration of the study may not fully capture the potential long-term effects of the therapies on fertility outcomes [14].

According to Isaiah IN et al., human sperm contains a lot of nutrient-rich components, it is ideal for the growth of bacteria. It could be contaminated by harmful microbes and normal skin flora. Research has indicated that bacteria in sperm can impact their quality. Physicians treat leukocytospermia and UTIs with a variety of antibiotics. However, research indicates that anti-infection medications have a negative effect on male reproductive potential and sperm parameters. When interpreting successful seminal fluid cultures, care was taken to account for factors such as the use of antibiotics, elevated colony counts of multiple strains in the seminal fluid, and increased colony counts of unique isolation in the semen. As a result, a typical misinterpretation of a genital tract infection is the presence of seminal bacteria [15].

Showell MG et al. concluded that OS harms DNA structure and speeds up cell apoptosis, impairing sperm function and hindering conception or embryonic development. Various antioxidant factors have demonstrated positive effects on sperm counts, pregnancy rates, and live birth rates in both *in vitro* and *in vivo* studies [16].

Walczak-Jedrzejowska et al. highlighted the importance of considering the dosage of antioxidant preparations. Since a mature sperm develops over approximately 72±4 days from spermatogonia, it suggests that in cases of OS, antioxidant doses should exceed the typical daily dose and be administered for at least 3 months. Further research is needed to establish the optimal active doses of antioxidants. While overdoing certain ingredients, such as vitamin A, can have adverse effects, non-prescription products typically contain safe amounts of individual components [17]. This study highlights the effects of bacteriospermia on male fertility, the significance of screening for bacterial infections before receiving treatment, and the function of antioxidant therapy in reducing DNA damage caused by OS.

## Conclusions

Incorporating bacteriospermia management and SDF reduction within a multidisciplinary treatment framework demonstrates the possibility of complete fertility care. Natural conception success emphasizes the need for individualized therapies, patient-centered care, and collaborative efforts to obtain favourable fertility results. The study also found a significant percentage of antibiotic resistance present in isolated strains. Additionally, the growing body of research emphasizes the importance of a strategy in the management of male infertility, providing hope and direction to both healthcare practitioners and couples for the management of fertility-related issues.
